# Physiological levels of the PTEN-PI3K-AKT axis activity are required for maintenance of Burkitt lymphoma

**DOI:** 10.1038/s41375-019-0628-0

**Published:** 2019-11-12

**Authors:** Franziska Gehringer, Stephanie Ellen Weissinger, Peter Möller, Thomas Wirth, Alexey Ushmorov

**Affiliations:** 1 Institute of Physiological Chemistry, University of Ulm, Ulm, Germany; 2 Institute of Pathology, University of Ulm, Ulm, Germany

**Keywords:** Oncogenesis, B-cell lymphoma

## Abstract

In addition to oncogenic MYC translocations, Burkitt lymphoma (BL) depends on the germinal centre (GC) dark zone (DZ) B cell survival and proliferation programme, which is characterized by relatively low PI3K-AKT activity. Paradoxically, PI3K-AKT activation facilitates MYC-driven lymphomagenesis in mice, and it has been proposed that PI3K-AKT activation is essential for BL. Here we show that the PI3K-AKT activity in primary BLs and BL cell lines does not exceed that of human non-neoplastic tonsillar GC DZ B cells. BLs were not sensitive to *AKT1* knockdown, which induced massive cell death in pAKT^high^ DLBCL cell lines. Likewise, BL cell lines show low sensitivity to pan-AKT inhibitors. Moreover, hyper-activation of the PI3K-AKT pathway by overexpression of a constitutively active version of AKT (myrAKT) or knockdown of *PTEN* repressed the growth of BL cell lines. This was associated with increased AKT phosphorylation, NF-κB activation, and downregulation of DZ genes including the proto-oncogene *MYB* and the DZ marker CXCR4. In contrast to GCB-DLBCL, PTEN overexpression was tolerated by BL cell lines. We conclude that the molecular mechanisms instrumental to guarantee the survival of normal DZ B cells, including the tight regulation of the PTEN-PI3K-AKT axis, also operate in the survival/proliferation of BL.

## Introduction

Burkitt lymphoma (BL) is the most common aggressive type of B cell lymphoma in children and also occurs in adults [1]. BL originates from germinal centre (GC) B cells [2], and is characterized by translocation of the proto-oncogene *MYC* under the control of immunoglobulin heavy or light chain loci [3]. The GC is divided in a dark zone (DZ) and a light zone (LZ). The DZ B cell proliferation and survival programme depends on expression of the transcription factors BCL6, FOXO1, and TCF3 and it is repressed by B cell receptor (BCR) and CD40 signalling [4]. In the LZ, survival signals via the BCR and CD40 [4] activate NF-κB, JAK-STAT, ERK, and PI3K-AKT pathways but simultaneously repress the DZ proliferation and survival programme [5–7].

In addition to MYC deregulation, BL maintains and is still dependent on the DZ survival and proliferation programme [2, 8–10]. This is reinforced by TCF3-stabilizing mutations, inactivating mutations of the TCF3 antagonist ID3, and CCND3 protein-stabilizing mutations [1, 11]. In accordance with a role of the DZ programme in BL, the LZ survival pathways NF-κB [2, 8, 12] and ERK/MAPK [13, 14] are attenuated in BL. Moreover, activation of NF-κB is inappropriate for MYC-driven B lymphomagenesis in a mouse model of BL, and induces apoptosis in BL cell lines [12].

There are contradictory data on the PI3K-AKT status in BL. A high PI3K-AKT activity has been proposed because constitutive PI3K activation facilitated MYC-driven B cell lymphomagenesis in mice [15]. Correspondingly, the mTORC2-dependent AKT^S473^ phosphorylation, which indirectly indicates PI3K activation, was detected in BL [11, 15]. Inactivating mutations of the purinoceptor P2RY8 are often observed in BL and these mutations have been suggested to result in activation of the PI3K-AKT pathway [16, 17]. In addition, it was supposed that activating TCF3 and inactivating ID3 mutations which increase tonic BCR-signalling might confer the high PI3K-AKT activity to BL [1, 11]. Moreover, BLs express high levels of *miR17-92HG*, which *inter alia* might attenuate PTEN expression [6, 11, 18].

However, the PI3K-AKT activity in BL has never been directly compared with normal DZ B cells. At the same time, there is a solid body of data contradicting the PI3K-AKT hyperactivation in BL. PI3K-PDPK1-dependent AKT^T308^ phosphorylation intensity in BL cell lines and BLs is detectable by immunohistochemistry (IHC) only in 21% of BL cases [19] and is much lower than in GC B cell like diffuse large B cell lymphomas (GCB-DLBCLs), which demonstrate high levels of PI3K-AKT activity often due to the lack of PTEN expression [14, 19, 20]. Moreover, the preferential nuclear localization even of non-mutated FOXO1 also contradicts the idea of AKT hyperactivation in BL [21]. In addition, the role of PTEN as tumour suppressor in BL has never been directly analysed in these studies by gain- or loss-of-function experiments.

Given that PI3K-AKT hyperactivation represses the DZ phenotype in normal B cells [6], it is conceivable that this pathway is also tightly controlled in BL. Consequently, we hypothesized that the PTEN-PI3K-PDPK1-AKT activity in BL must be maintained at levels of DZ B cells, to prevent extinguishing of the GC DZ programme that BLs are addicted to.

## Methods

Additional and detailed information on methods are provided in the Supplementary Data.

### Cell lines

BL cell lines (Ramos, BL-41, Namalwa, Daudi, Jiyoye, Raji) and DLBCL cell lines (BJAB, SU-DHL-5, WSU-NHL, OCI-Ly1, WSU-DLCL2, Karpas-422, HT, OCI-Ly19, SU-DHL-4, and DoHH2) were purchased from DSMZ, Braunschweig, Germany. The culture conditions, analysis of cell line identity, and mycoplasma status were analysed as described in Supplementary Methods.

### GC DZ B cell isolation

Tonsillar GC DZ B cells were isolated from tonsils of three 29–35 years old patients undergoing tonsillectomy at the Department of Otorhinolaryngology, Head and Neck Surgery, University of Ulm, Germany. The written informed consent was obtained. CD19^+^/IgD^−^/CD38^hi^/CXCR4^hi^/CD86^lo^ cells representing GC DZ B cells [10] were isolated as described in Supplementary Methods.

### Tissue samples and immunohistochemistry (IHC)

Nine BL and nine GCB-DLBCL samples were drawn from our archive of frozen and formalin-fixed paraffin-embedded tissues. The diagnoses were based on histologic, immunohistologic, and molecular diagnostic grounds according to the WHO [22]. Samples were pseudonymised according to the German law for correct usage of archival tissue for clinical research [23]. Approval for this procedure was obtained from the local Ethics Committee (vote for usage of archival human material 03/2014) and was in compliance with the ethical principles of the WMA Declaration of Helsinki. Immunostaining using anti-pAKT^T308^ (Abcam, #38449) was performed and analysed as described in Supplementary Methods.

### Vectors and lentiviral transduction

The cell lines were transduced as described in Supplementary Methods. For expression of the shRNA constructs we used the pRSI12-U6-sh-UbiC-TagRFP-2A-Puro lentiviral vector (BioCat, Heidelberg, Germany). For gene expression we used the SF-LV-cDNA-eGFP vector as described in Supplementary Methods.

### Immunoblot and qRT-PCR

Immunoblot and qRT-PCR were done as described in Supplementary Methods.

Primer sequences and antibodies are listed in Supplementary Methods.

### Flow cytometry, cell sorting, and cell viability analysis

Growth dynamics of cell lines transduced with lentiviral vectors expressing fluorescent markers RFP or GFP were monitored by flow cytometry (FACSCanto II, BD Biosciences, San Jose, CA, USA). For biochemical analysis, RFP^+^ or GFP^+^ cells were sorted using a FACSAria (BD Biosciences) by the Core Facility “Fluorescent Activated Cell Sorting” (Medical Faculty of Ulm, Germany) or by the S3e Cell Sorter (Bio-Rad, Hercules, CA, USA). Cell death was measured by Annexin V-FITC/PI staining as we described previously [24]. CXCR4 surface staining (anti-CXCR4-APC, Thermo Fisher, #17472441) was measured using the flow cytometer FACSCanto (BD Biosciences). The sensitivities of the cell lines to the AKT inhibitor AZD5363 were assessed by MTT test. IC50 was calculated by fitting the data points to a nonlinear regression curve using GraphPad Prism (GraphPad Software, San Diego, CA).

### Statistical analysis

The data were analysed by two-tailed Student’s *t*-test analysis (Microsoft Excel) and by Mann-Whitney U test using “Mann–Whitney U Test Calculator” with help of Social Science Statistics Calculator (socscistatistics.com, 13.09.2018).

## Results

### PI3K-AKT activity of BL cell lines does not exceed that of GC DZ B cells and is much lower than of PTEN-negative GCB-DLBCL cell lines

**Fig. 1:**
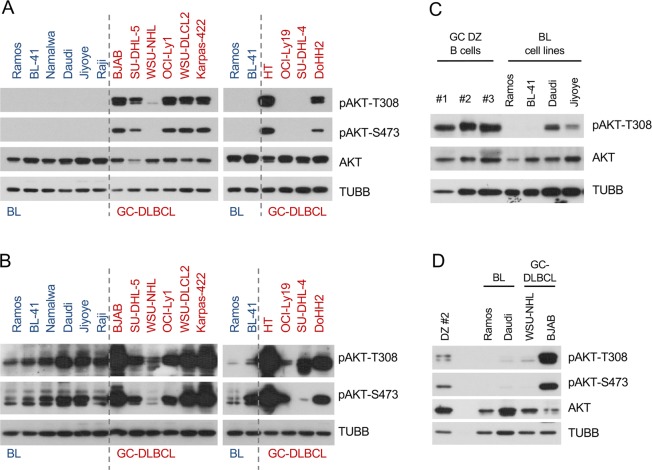
Low PI3K-AKT activity in BL in comparison with GCB-DLBCL cell lines and tonsillar GC DZ B cells. **a**, **b** Expression and phosphorylation status AKT in BL and GCB-DLBCL cell lines was analysed by immunoblot. TUBB served as loading control. A representative of two independent experiments is shown. **a** Short exposure. **b** Long exposure. **c** Immunoblots of pAKT^T308^ and total AKT in three FACS-sorted tonsillar GC DZ B cells as well as two BL cell lines with lowest and highest pAKT^T308^ expression levels. **d** Expression and phosphorylation status of AKT in FACS-sorted tonsillar GC DZ B cells (sample #2 as shown in **c**) compared with BL and GCB-DLBCL cell lines with lowest and highest pAKT^T308^ levels was analysed by immunoblot

In an attempt to shed light on the contradictory observations regarding the role of the PI3K-AKT pathway in BL, in a first approach we compared the PI3K-AKT activation status in BL with GCB-DLBCL cell lines, the oncogenic programme of which is known to often depend on PI3K-AKT hyperactivation (pAKT^high^) [25, 26]. We included seven (pAKT^high^) GCB-DLBCL cell lines with strong (BJAB, SU-DHL-5, OCI-Ly1, WSU-DLBCL2, Karpas-422, HT, DoHH2) and three with weak (WSU-NHL, OCI-Ly19, SU-DHL-4) (pAKT^low^) AKT activity and compared the PI3K-PDPK1-dependent AKT^T308^ and auxiliary mTORC2-dependent AKT^S473^ phosphorylation signals [27, 28] with that of six BL cell lines. At short exposure time, pAKT^T308^ and pAKT^S473^ signals were detected in all pAKT^high^ GCB-DLBCL cell lines (Fig. 1a) but in none of BLs. pAKT^T308^ and pAKT^S473^ signals were detected in BL cell lines only at longer exposure time, when the signals of pAKT^high^ GCB-DLBCLs were out of the dynamic range (Fig. 1b).

We then evaluated the PI3K-AKT activity in BL cell lines in comparison to its normal counterpart, CD19^+^/IgD^−^/CD38^hi^/CXCR4^hi^/CD86^lo^ human tonsillar GC DZ B cells. Given that expression of the LZ marker CD86 in humans decreases slower in the process of LZ to DZ transition than CD83 [10], we chose CD86 to discriminate remnants of LZ-specific PI3K-AKT activation (Fig. S1a). The authenticity of the isolated DZ B cells was confirmed by analysis of the cell cycle distribution (30.6% in S- and G_2_/M-phases and clearly visible G_2_/M peak (9.2 %) (Fig. S1b) [10]. We compared non-neoplastic tonsillar GC DZ samples of three different patients with BL cell lines that previously demonstrated the lowest and highest pAKT^T308^ signals (two of highest and two of lowest expression, Fig. 1a, b). This analysis revealed that all three GC DZ B cell preparations demonstrated similar levels of pAKT^T308^ which was higher than in any of the BL cell lines (Fig. 1c). By including two GCB-DLBCL cell lines with highest and lowest pAKT^T308^ expression into the analysis (Fig. 1a, b), we found that the levels of AKT phosphorylation in pAKT^high^ GCB-DLBCL cell lines exceed that of GC DZ B lymphocytes (Fig. 1d).

### Primary BLs maintain the physiological PI3K-AKT activation status

**Fig. 2:**
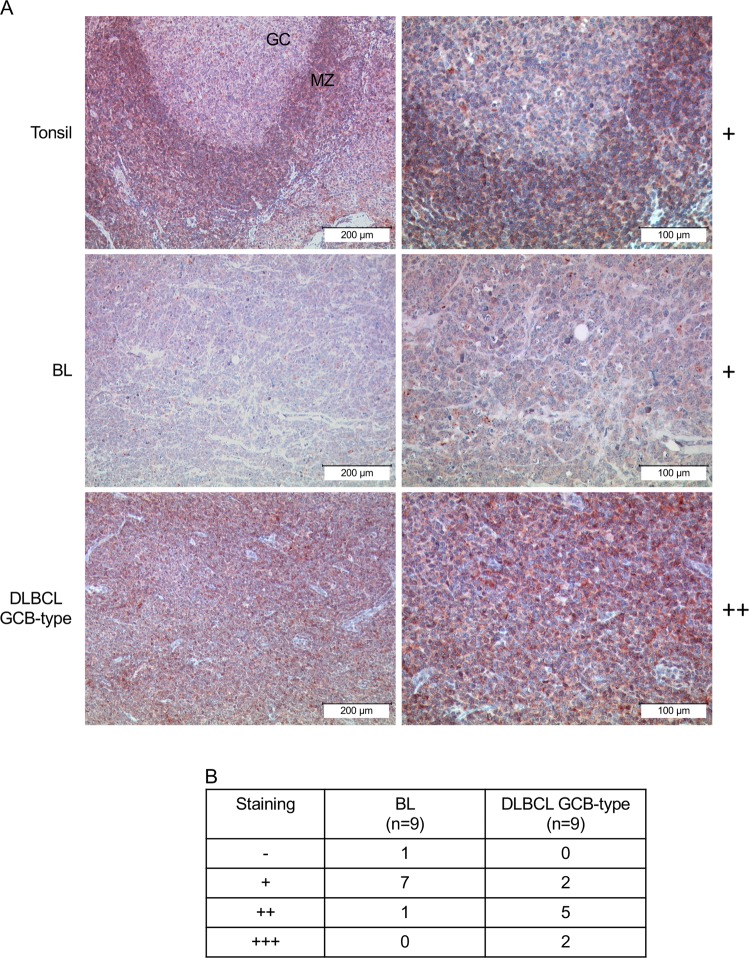
Physiological PI3K-AKT activation in BL. **a** Immunohistochemical detection of pAKT^T308^. Normal tonsillar GC B cells (GC) are weakly positive (+) while follicular mantle B cells (MZ) are strongly positive (+++). In BL pAKT^T308^ was weakly positive in most cases. The majority of GCB-DLBCL was clearly positive (++). Left column: Overview. Scale bar represents 200 µm. Right column: Details. Scale bar represents 100 µm. **b** Table gives details of all cases examined

Finally, we compared the AKT^T308^ phosphorylation status in non-neoplastic tonsils with BL and GCB-DLBCL tumours using an antibody, which was previously described to specifically detect pAKT^T308^ by IHC [29] (Fig. S2). Overall, the phosphorylation intensity in BL was weak and comparable with GC B cells, whereas most GCB-DLBCL cases demonstrated medium or strong, but heterogeneous pAKT^T308^ staining (Fig. 2a, b).

Thus, in most cases BL maintains PI3K-AKT activation comparable to non-neoplastic GC B cells.

### BL cell lines are less sensitive to AKT inactivation than pAKT^high^ GCB-DLBCL cell lines

**Fig. 3:**
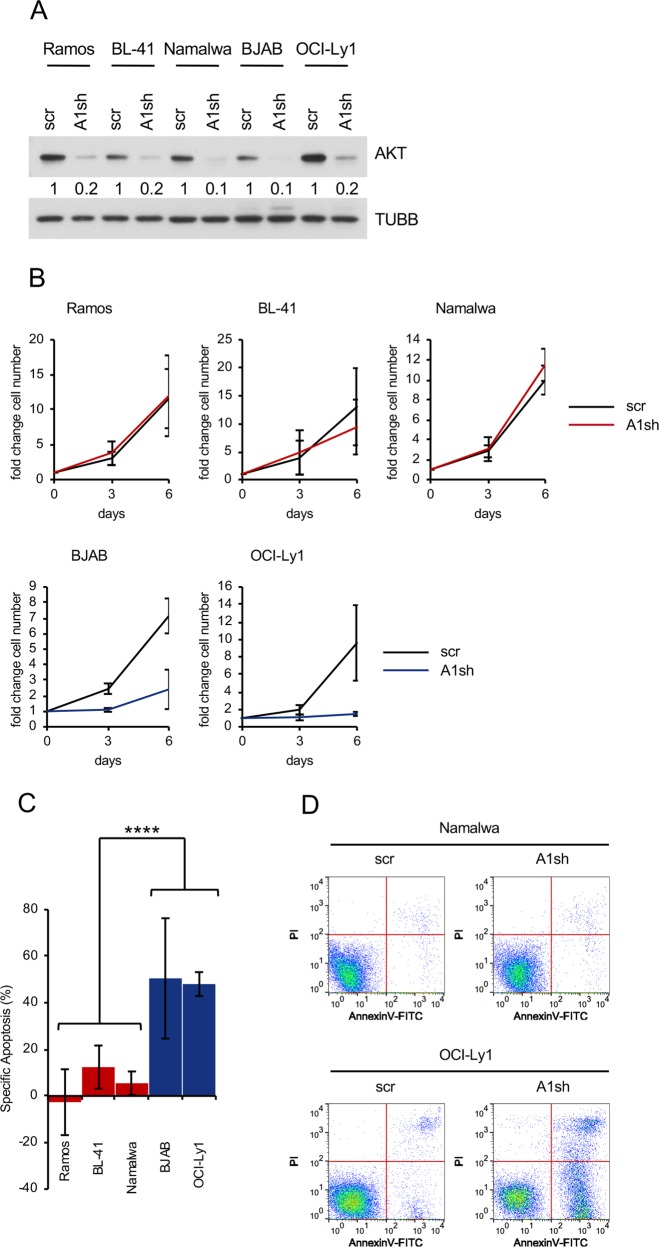
BL cell lines are less sensitive to *AKT1* knockdown than pAKT^high^ GCB-DLBCL cell lines. BL and GCB-DLBCL cell lines were transduced with vectors expressing *AKT1* shRNA (A1sh) vs. scrambled (scr) control. **a** Knockdown efficiencies of AKT1sh vs. scr control. Transduced cells were FACS-sorted 5 days post transduction and total AKT expression was analysed. AKT expression was quantified using ImageJ software. A representative of two independent experiments is shown. **b** Cells were FACS-sorted 4 days post transduction, followed by cell counting using a cell viability analyser. Initial number of cells was set as 1. Data are shown as mean ± SD (*N* = 3). **c** Cell death analysis of cells expressing A1sh or scr. Transduced cells were sorted 4 days post transduction, and incubated in complete medium for 6 days followed by Annexin V-FITC/PI staining. Specific Apoptosis (SA) was calculated as SA (%) = 100 × (AE − AC)/(100 − AC), where AE equals the percentage of apoptotic cells in the experimental group and AC equals the percentage of apoptotic cells in the control group. Data are shown as mean ± SD (N = 3). The data were analysed by two-sided T-test. ****P < 0.0001. **d** Representative dot-plot images as shown in **c**

In DLBCL, a high AKT phosphorylation status correlates with the sensitivity to genetic *AKT* depletion [30, 31]. Even the knockdown of *AKT1* alone, the most highly expressed of three AKT isoforms, induces massive cell death in pAKT^high^ DLBCLs [30]. At the same time, normal mouse B cells require minimal AKT activity. Only simultaneous knockout of the two major *AKT* genes *AKT1* and *AKT2* is toxic for them [32]. Therefore, the sensitivity to *AKT1* knockdown might be used as a criterion of oncogenic AKT dependency. The *AKT1* shRNA reduced total AKT levels to 10–20 % in comparison with the scrambled shRNA in BL and DLBCL cell lines (Fig. 3a). In pAKT^high^ DLBCL cell lines *AKT1* knockdown strongly decreased the number of live cells (Fig. 3b), which was associated with massive apoptosis (Fig. 3c, d). In contrast, in BL *AKT1* knockdown did not influence cell growth and did not induce significant cell death (Fig. 3b, d). We conclude that BL is much less dependent on PI3K-AKT activation than pAKT^high^ GCB-DLBCL.

### BL cell lines are less sensitive to AKT inhibitors than GCB-DLBCL cell lines

**Fig. 4:**
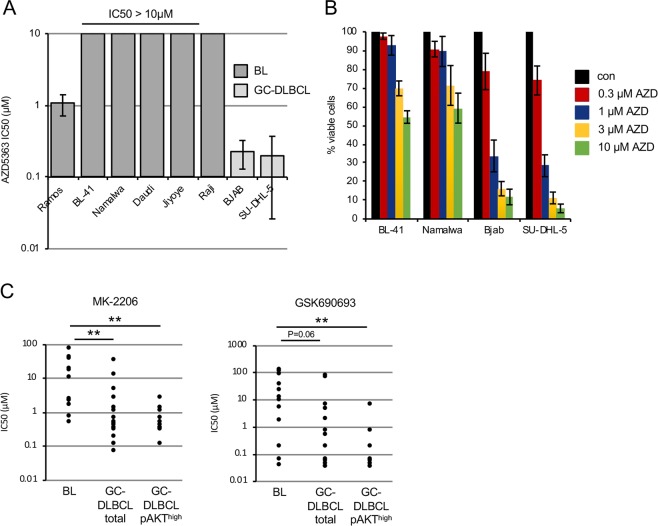
Low sensitivity of BL cell lines to AKT inhibitors. **a** Sensitivity of BL and GCB-DLBCL cell lines to AZD5363 (AZD). Cells were exposed to various concentrations of AZD for 5 days. Cell viability was assessed using MTT assay and IC50 values were calculated using GraphPad Prism software. Data are shown as mean ± SD (*N* = 3). **b** Percentage of viable BL or GCB-DLBCL cells 5 days post treatment with AZD5363. Cells were counted using a cell viability analyser. Initial number of cells was set as 100%. Data are shown as mean ± SD (*N* = 3). **c** Sensitivity of BLs with reported t(8;14) MYC translocations, not otherwise specified GCB-DLBCL, and AKT^high^ GCB-DLBCL cell lines to MK-2206 or GSK690693. IC50 values were obtained from cancerrxgene.org and analysed using Mann–Whitney U test with help of Social Science Statistics Calculator (socscistatistics.com). MK-2206: BL vs. total GCB-DLBCL **p = 0.004. BL vs. pAKT^high^ GCB-DLBCL **p = 0.001. GSK690693: BL vs. total GCB-DLBCL ns, *p* = 0.060. BL vs. pAKT^high^ GCB-DLBCL **p = 0.009. Maximum screening concentration: MK-2206 = 4 μM; GSK690693 = 10.2 μM

Pharmacological inhibition of the PI3K pathway has been suggested as treatment option in BL [15]. However, our observations of the maintenance of PI3K-AKT activity at physiological levels and insensitivity to the *AKT1* knockdown contradict this assumption. Therefore, we measured the sensitivities of BL cell lines to the pan-AKT inhibitor AZD5363 in comparison with pAKT^high^ GCB-DLBCL cell lines, which are highly sensitive to AZD5363 in vitro and in vivo [26]. All BLs were much less sensitive to AZD5363 than GCB-DLBCL cell lines as measured by MTT viability assay (Fig. 4a) and by cell counting (Fig. 4b). With exception of Ramos, in all BL cell lines the IC_50_ value was higher than 10 µM, and therefore 50-fold higher than of GCB-DLBCL cell lines.

In addition, we mined data on the sensitivity of BL and GCB-DLBCL cell lines to the pan-AKT inhibitors MK-2206 and GSK690693 from the Genomics of Drug Sensitivity in Cancer database (http://www.cancerrxgene.org/, 09-13-2018). We compared the sensitivities of BL cell lines with all GCB-DLBCL cell lines listed in the database and separately with pAKT^high^ GCB-DLBCL [25, 26]. BL cell lines were significantly less sensitive to the pan-AKT inhibitors than pAKT^high^ GCB-DLBCLs (Table S1 and Fig. 4c). Of note, differences in the sensitivity of GCB-DLBCL and BL cell lines to the allosteric inhibitor MK-2206 were remarkably higher than in the case of treatment with the ATP-competitive inhibitor GSK690693. This might be explained by the higher selectivity of allosteric inhibitors [33]. GSK690693 inhibits in addition to AKTs also DAPK3, PAK4-6, PKA, and different PKCs at similar concentrations [34]. AZD5363 inhibits P70S6K and PKA at the same concentration as AKTs, and already at 1 µM ROCK1, MKK1, MSK1, MSK2, PKCγ, PKGα, PKGβ, PRKX, RSK2, and RSK3 were inhibited by more than 75% [35]. Thus, we found that most BL cell lines are not sensitive to the AKT inhibitors at concentrations at which they predominantly inhibit AKT kinases.

### AKT hyperactivation represses the DZ phenotype in BL cell lines

**Fig. 5:**
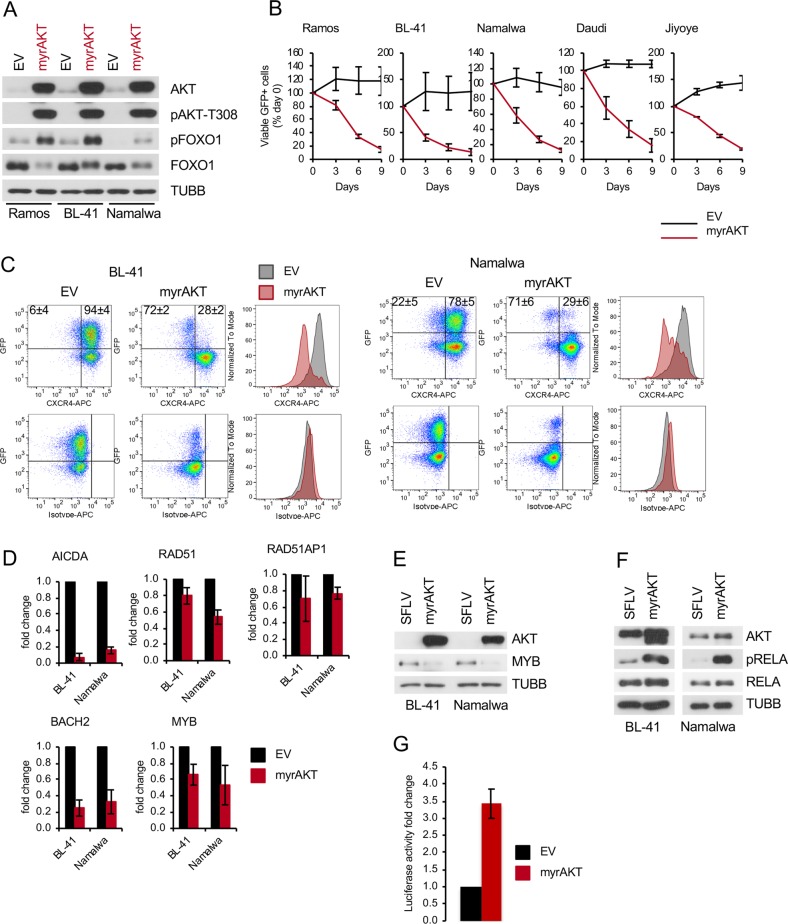
myrAKT overexpression is toxic for BL cell lines. **a**–**f** BL cell lines were transduced with lentiviral plasmids expressing constitutively active myrAKT or empty vector (EV). **a** Transduced cells were FACS-sorted 4 days post transduction and analysed for expression of total AKT and FOXO1 and pAKT^T308^ and pFOXO1^T24^ levels. TUBB served as loading control. A representative of two independent experiments is shown. **b** Percentage of transduced cells was measured every 3 days using flow cytometry. First measurement was performed 4 days post transduction and the percentage of GFP^+^ cells was set as 100. Data are shown as mean ± SD (*N* ≥ 3). **c** myrAKT downregulates CXCR4. BL cell lines expressing myrAKT or EV were stained with CXCR4-APC or isotype control 5–7 days post transduction. Dot-plots show percentages of CXCR4^+^/GFP^+^ and CXCR4^−^/GFP^+^ cells. For histograms, only transduced GFP^+^ cells were included. Data are shown as mean ± SD (*N* = 3). **d** Downregulation of critical GC genes after myrAKT overexpression. Transduced cells were sorted 3 days post transduction and RNA expression levels were analysed by qRT-PCR. qRT-PCR data were quantified by the 2^−ΔΔCT^ method. Data are shown as mean ± SD (*N* = 3). The data were analysed by two-sided *T*-test. For all genes and cell lines *p* < 0.05 with exception of BL-41 RAD51AP1 *p* = 0.135. **e** MYB expression in sorted GFP^+^ cells was analysed by immunoblot 3 days post transduction with myrAKT or EV. TUBB served as loading control. A representative of two independent experiments is shown. **f** myrAKT activates NF-κB. BL cell lines expressing myrAKT or EV were FACS-sorted 3 days post transduction and pRELA^S536^ and total RELA were analysed by immunoblot. TUBB served as loading control. A representative of two independent experiments is shown. **g** Luciferase reporter assay. Namalwa cells stably expressing a NF-κB-dependent luciferase reporter (3 × κB.luc) [60] were transduced with a vector expressing myrAKT. GFP^+^ cells were sorted 4 days post transduction. Luminescence was measured as described in Supplementary Methods. Data are shown as mean ± SD (*N* = 3)

Since we found that the PI3K-AKT activity in BLs does not exceed that of non-neoplastic GC DZ B cells, we asked whether BLs would tolerate an increase in PI3K-AKT activity beyond these physiological levels. To this end we transduced BL cell lines with lentiviral vectors expressing GFP and a constitutively active version of AKT1 (myrAKT) or the empty vector control and monitored the dynamic of the GFP^+^ cell population and the phosphorylation status AKT and AKT-inactivation target FOXO1. FOXO1 has been shown to be essential for the growth of BL [21]. MyrAKT overexpression enhanced the levels of PDPK1-dependent AKT^T308^ and AKT-dependent FOXO1^T24^ phosphorylation, and decreased total FOXO1 levels (Fig. 5a). This was associated with a rapid decrease of the GFP^+^ population (Fig. 5b) whereas the control vector did not affect the growth of the cell lines.

Given that strong PI3K activation represses the DZ programme in mouse GC B cells [6], we checked whether myrAKT overexpression downregulates the essential DZ marker and FOXO1 target gene CXCR4 [5]. Decrease of the CXCR4 expression was observed only in GFP^+^ cells transduced with myrAKT, but not by the control vector (Fig. 5c). In contrast, cells that were stained with an isotype control antibody did not show any changes. Since CXCR4 is a target of the proto-oncogene MYB [36–38], which is highly expressed in GC DZ B cells and is essential for the survival of BL cell lines [39, 40], we analysed the influence of AKT hyperactivation on MYB expression. MyrAKT downregulated MYB expression at mRNA (Fig. 5d) and protein levels (Fig. 5e). Moreover, the transcription of several DZ B cell signature genes including *AICDA*, *RAD51*, *RAD51AP1*, and *BACH2* was also repressed by AKT hyperactivation (Fig. 5d).

AKT directly phosphorylates and activates IKKA and IKKB kinases, which in turn phosphorylate p65/RELA resulting in NF-κB activation [41, 42]. NF-κB signalling is attenuated in BL cell lines and NF-κB activation induced apoptosis in these cells [12]. NF-κB signalling is also repressed in the DZ, whereas LZ GC B cells show activation of the NF-κB signalling cascade [43]. Therefore, we measured NF-κB activation in BL cell lines after overexpression of myrAKT and observed increased IKK-dependent RELA^S536^ phosphorylation (Fig. 5f). Moreover, using a NF-κB-responsive luciferase reporter construct we confirmed activation of the NF-κB-dependent transcription by AKT overexpression (Fig. 5g).

We conclude that hyperactivation of AKT is incompatible with maintenance of the DZ programme and ultimately survival of BL cell lines.

### PTEN expression is essential for BL maintenance

**Fig. 6:**
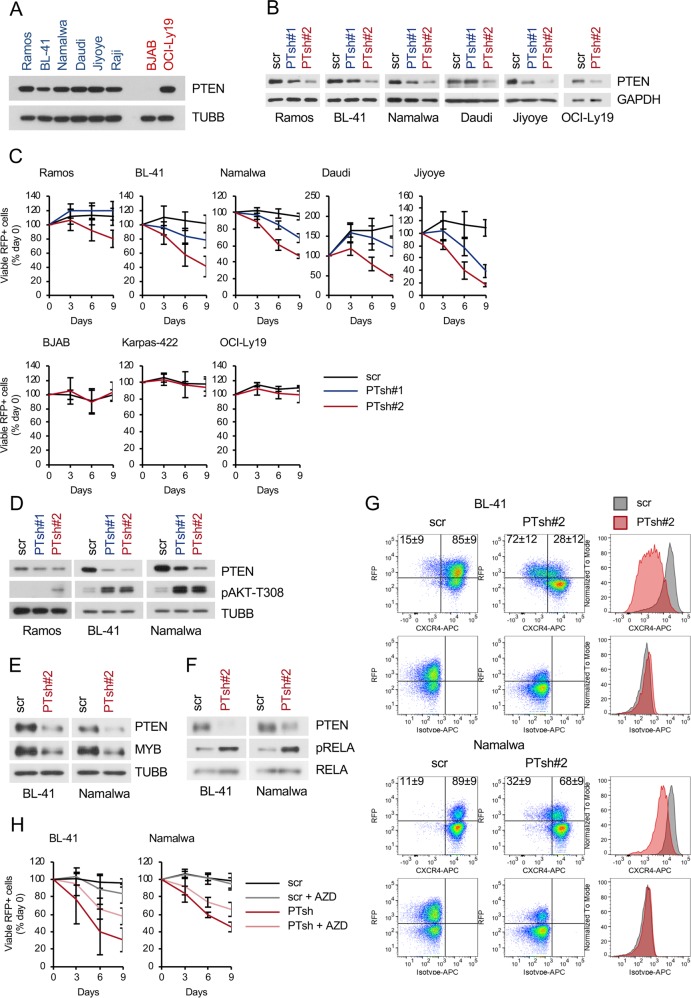
PTEN expression is essential for BL cell lines. **a** Expression of PTEN in BL was analysed by immunoblot. GCB-DLBCL cell lines were included as controls. A representative of two independent experiments is shown. **b**–**h** BL cell lines and/or GCB-DLBCL cell lines were transduced with vectors expressing *PTEN* shRNAs (PTsh#1, PTsh#2) or scr control. **b** Knockdown efficiencies of PTsh vs. scr control in BL cell lines and PTEN-positive GCB-DLBCL cell line OCI-Ly19. Transduced cells were selected using 4 µg/mL puromycin or FACS-sorted and PTEN expression was analysed. A representative of two or three independent experiments is shown. **c** Growth dynamics of transduced BL and GCB-DLBCL cell lines. The percentage of RFP^+^ cells was measured every 3 days using flow cytometry. First measurement was performed 4 days post transduction and the percentage of RFP^+^ cells was set as 100. Data are shown as mean ± SD (*N* ≥ 3). **d** Transduced cells were FACS-sorted 4–5 days post transduction and PTEN and pAKT^T308^ levels were analysed using immunoblot. A representative of two independent experiments is shown. **e** MYB expression levels in transduced and FACS-sorted BL cell lines expressing PTsh#2 or scr control were analysed by immunoblot 3 days post transduction. A representative of two independent experiments is shown. **f** BL cell lines expressing PTsh#2 or scr control were FACS-sorted 4 days post transduction and pRELA^S536^ and total RELA were analysed by immunoblot. TUBB served as loading control. A representative of two independent experiments is shown. **g** PTsh#2 downregulates CXCR4. BL cell lines expressing PTsh#2 or scr control were stained with CXCR4-APC or isotype control 4–7 days post transduction. Dot-plots show percentages of CXCR4^+^/RFP^+^ and CXCR4^−^/RFP^+^ cells. For histograms, only transduced RFP^+^ cells were included. Data are shown as mean ± SD (*N* = 3). **h** AZD5363 (AZD) treatment partially rescues BL cell lines from the growth inhibitory effect of *PTEN* knockdown. BL cell lines were treated with 1 µM AZD 4–5 days post transduction with PTsh#2 and the percentage of RFP^+^ cells was measured every 3 days using flow cytometry. The percentage of RFP^+^ cells at first measurement was set as 100. Data are shown as mean ± SD (N = 3)

Inactivation of PTEN, a negative regulator of PI3K-AKT, was suggested to contribute to PI3K-AKT hyperactivation in BL [11], although functional consequences of *PTEN* knockdown have never been investigated in BL cell lines. We found PTEN protein in all BL cell lines, whereas 50 % of GCB-DLBCLs displayed PTEN loss (Figs. 6a and S3a), closely representing the percentage of PTEN loss reported in GCB-DLBCL patient samples [25]. To clarify the role of PTEN in BL, we transduced BL and GCB-DLBCL cell lines with vectors co-expressing PTEN shRNAs together with RFP. Two shRNA constructs, which showed different efficiencies of PTEN downregulation, were employed (Fig. 6b). In the competitive growth assay we observed significant time-depended decrease of the RFP^+^ cell population in all BL cell lines transduced with PTEN shRNA-expressing vectors (Fig. 6c). Of note, the *PTEN* knockdown efficiencies of the constructs correlated with their cytotoxic effects (Fig. 6b, c). As controls, two PTEN-negative (BJAB and Karpas-422) and one PTEN-positive (OCI-Ly19) GCB-DLBCL cell line were included. In contrast to BL, *PTEN* knockdown did not affect the growth of GCB-DLBCLs, independent of the PTEN status (Fig. 6b, c). Importantly, the shRNA-mediated *PTEN* knockdown increased the pAKT^T308^ signal (Fig. 6d) and increased the levels of pFOXO1^T24^ (Fig. S3b). Consistent with myrAKT overexpression, *PTEN* knockdown decreased MYB (Fig. 6e) and CXCR4 (Fig. 6g) protein expression and increased RELA^S536^ phosphorylation and the transcriptional activity of a NF-κB-dependent reporter (Figs. 6f and S3c).

To clarify the role of AKT activation in the cytotoxic effect of *PTEN* knockdown, we treated cells transduced with a *PTEN*-targeting shRNA with the pan-AKT inhibitor AZD5363. AZD5363 could partially protect BL-41 and Namalwa cell lines from the anti-proliferative effect of *PTEN* knockdown (Fig. 6h).

Therefore, PTEN contributes to BL survival at least in part by attenuating the PI3K-AKT activity.

### PTEN overexpression does not influence the survival of BL

**Fig. 7:**
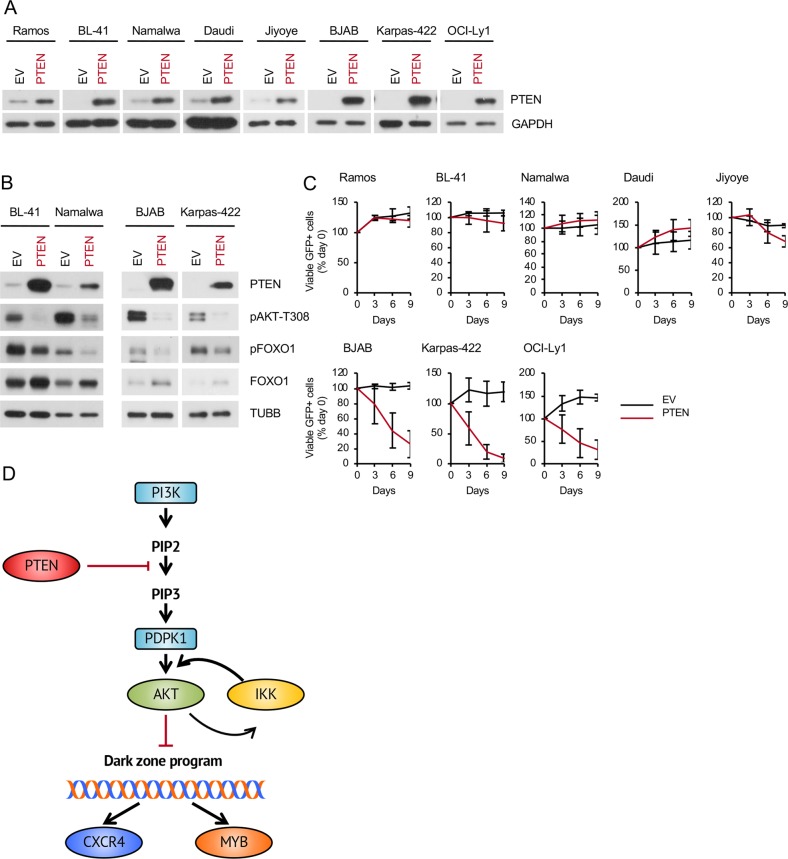
PTEN overexpression induces growth inhibition in GCB-DLBCL but not BL cell lines. **a**–**c** BL and GCB-DLBCL cell lines were transduced with vectors expressing PTEN vs. empty vector (EV) control. **a** Transduction efficiencies were ranging from 11% to 100%. Transduced cells were lysed 4–5 days post transduction and PTEN overexpression was confirmed by immunoblot. GAPDH served as loading control. A representative of two independent experiments is shown. **b** Transduced cells were FACS sorted 4 days post transduction and analysed for expression of PTEN, FOXO1, pAKT^T308^, and pFOXO^T24^. TUBB served as loading control. A representative of two independent experiments is shown. **c** Growth dynamics of transduced BL and GCB-DLBCL cell lines. The percentage of GFP^+^ cells was measured every 3 days using flow cytometry. First measurement was performed 4–5 days post transduction and the percentage of GFP^+^ cells was set as 100. Data are shown as mean ± SD (*N* = 3). **d** Illustration of the maintenance of physiological mechanisms of PI3K-AKT regulation in BL. BLs maintain the original PI3K-AKT and IKK-NF-κB activation status to prevent extinguishing of the DZ programme, including CXCR4 and MYB. Part of the image is adapted from Motifolio Drawing Toolkits (www.motifolio.com)

Downregulation of *PTEN* by *miR17-92HG* was suggested to be an oncogenic factor in BL [1], but the role of PTEN as tumour suppressor in BL has never been addressed by a gain-of-function experiment. To clarify this issue, we overexpressed PTEN in BL cell lines. Three PTEN-negative GCB-DLBCL cell lines (BJAB, Karpas-422, and OCI-Ly1), which do not tolerate re-expression of PTEN [25] were used as positive controls (Fig. 7a). As expected, PTEN overexpression decreased pAKT^T308^ and pFOXO1^T24^ signal intensities, and increased expression of FOXO1 protein in both, BL and GCB-DLBCL cell lines (Fig. 7b). Interestingly, PTEN overexpression suppressed the growth of all GCB-DLBCL cell lines, whereas BL cell lines were not affected (Fig. 7c).

We conclude that PTEN does not act as tumour suppressor in BL, even at supra-physiological levels.

## Discussion

We found that the PI3K-AKT activity in primary BL and BL cell lines does not exceed that of human GC DZ B cells. The lower PI3K-AKT activity in BL cell lines was associated with lower sensitivity to genetic and pharmacological AKT inhibition. In addition, we show that AKT hyper-activation in BL cell lines results in repression of the DZ-specific differentiation programme including the DZ marker CXCR4 and the proliferation and survival gene MYB. AKT hyperactivation was also associated with activation of NF-κB. Finally, we show the critical role of the PI3K repressor PTEN in controlling the PI3K-AKT and IKK-NF-κB activities in BL (Fig. 7d).

Here, for the first time we directly compared the PI3K-PDPK1-dependent AKT^T308^ and mTORC2-dependent AKT^S473^ phosphorylation intensities in BL cell lines with human tonsillar GC DZ B cells. We found that BLs maintain physiological PI3K-AKT activity levels comparable to non-neoplastic GC DZ B cells. In addition, we confirmed the low PI3K-AKT activation status in BL in comparison with pAKT^high^ GCB-DLBCL cell lines [14, 20] and lower pAKT^T308^ expression in primary BL in comparison with GCB-DLBCL, as it was assessed by IHC [19].

Our data apparently contradict the commonly accepted view of BL as an archetype of oncogenic coupling of MYC and PI3K-AKT activity [1, 11, 15]. This concept is based on the potentiating effect of PI3K activation on MYC-dependent B cell lymphomagenesis in a mouse model [15]. The presence of FOXO1-activating mutations targeting the AKT-recognition motif in B lymphomas derived from this model represents strongest evidence of the similarity with BL. This was interpreted as an adaptive mechanism, which maintains FOXO1 activity despite oncogenic AKT activation [15]. Nevertheless, there is a principal difference between the mouse model and BL, which contradicts the concept of the oncogenic PI3K-AKT activation. In BL, FOXO1 does not require activating mutations to be localised in the nucleus [21]. Moreover, there is no evidence of a role of FOXO1-activating mutations in BL progression. The clinical outcome of BL does not depend on the FOXO1 mutational status and there is no increase in the frequency of FOXO1 mutations in relapsed cases [44]. This is in clear contrast to DLBCL in which FOXO1 mutations are associated with poor outcome [45, 46] and their frequency strongly increases in refractory or relapsed cases [47]. Nevertheless, we believe that PI3K-AKT activation may play a role in MYC-dependent B lymphomagenesis. The frequent association of BL with EBV infection, which activates PI3K-AKT signalling, indicates a role of PI3K-AKT activation at early stages, but the establishing of BL apparently requires repression of the EBV programme, including PI3K-AKT- and NF-κB–activating LMP genes [8, 14, 48]. This view is supported by the observation that FOXO1 mutations are found at a significantly higher frequency in endemic BL cases compared with sporadic BL [44]. Thus, frequent FOXO1-activating mutations, which confer resistance to the AKT-dependent inactivation, might be remnants of the adaptive response to the initial transitory PI3K-AKT activation.

In accordance to the physiological PI3K-AKT activity, we revealed low sensitivity of BLs to AKT inhibitors. These data are in line with the reported low sensitivity of the cell lines Raji, Ramos, and Daudi to the AKT inhibitor MK-2206 [49]. Similarly, BL cell lines appeared to be less sensitive to the AKT inhibitor GSK690693 than other NHLs and acute lymphoblastic leukemia cell lines [50].

To understand a role of the maintenance of PI3K-AKT activity at physiological levels, we used two different genetic models for pathway hyperactivation. In one approach we overexpressed the constitutively active version of AKT (myrAKT) in BL cell lines. MyrAKT mediates a supra-physiological activation of this pathway and induces apoptosis even in BCR-ABL1-dependent B cell acute leukemia in which constitutive activation of PI3K-AKT is an important oncogenic event. Our data support the assumption of the sensitivity of B-lineage neoplasia to very high levels of PI3K-AKT activation [51]. It is conceivable that known mechanisms of AKT hyperactivation-induced cell death including activation of TP53 and repression of oxidative phosphorylation [52] are also active in BL. Interestingly, we observed the repression of DZ genes after AKT hyperactivation, which resembles the effect of constitutive PI3K activation in GC B cells in mice [6]. This is in line with the higher PI3K-AKT activity in LZ B cells than DZ B cells [5, 6].

We found that overexpression of myrAKT in BL was associated with IKKB- and IKKA-dependent RELA phosphorylation and NF-κB activation. Given that AKT activates NF-κB by IKK phosphorylation [53], this finding is not surprising. Interestingly, the NF-κB signature is repressed in BL in comparison with ABC- and even with GCB-DLBCL [9]. Given that active PI3K-AKT can induce the NF-κB pathway, this is further arguing against the hypothesis of AKT-hyperactivation in BL. The low NF-κB activity might facilitate BL survival because NF-κB activation represses MYC-driven lymphomagenesis and is toxic for BL cell lines [12, 54]. Importantly, NF-κB can potentiate PI3K-AKT activity, e.g., by suppression of PTEN [55] creating a self-amplifying circuit. We conclude that maintenance of the PI3K-AKT activity at physiological levels represents the addiction of BLs to the GC DZ B cell survival and proliferation programme.

We identified PTEN as an essential survival factor regulating the PI3K and IKK activities in BL. This finding again contradicts the common view of PTEN as a tumour suppressor in BL [1, 18]. Although *PTEN* mutations are rare in BL in comparison with GCB-DLBCLs [11, 25, 56, 57] and its expression can be identified by immunohistochemistry in most BL samples [58], it was suggested that *miR-17-92HG*-dependent PTEN repression activates PI3K-AKT in BL. However, this hypothesis has never been directly experimentally addressed. Moreover, fine-tuning of MYC as the main function of *miR-17-92HG* was described in another study [59]. Given that microRNAs act rather as rheostat than as a switch in regulation of protein expression, one might assume that *miR-17-92HG* is involved in tight regulation of PTEN in BL, but our data on the insensitivity of BL cell lines to PTEN overexpression also question this assumption.

We revealed obvious similarities between the effects of myrAKT overexpression and *PTEN* knockdown. Both treatments increased AKT and IKK-NF-κB activities and decreased expression of the DZ marker CXCR4 and proto-oncogene MYB. In fact, we were able to reproduce the negative effects of *PTEN* knockdown and PI3K hyperactivation on DZ B lymphocytes [6] in BL cell lines. Of note, the dependency on PTEN expression is not unique. Genetic depletion of *PTEN* also inhibits leukemogenesis and induces apoptosis in BCR1-ABL-dependent pre-B-ALL [52].

We conclude that the PTEN-PI3K-AKT axis activity needs to be tightly controlled and maintained at physiological level in the oncogenic programme of BL.

## Supplementary information

Supplemental Material
