# Supplementary material for: Physiological levels of the PTEN-PI3K-AKT axis activity are required for maintenance of Burkitt lymphoma
Source: Leukemia. 2019 Nov 12;34(3):857–71. doi: 10.1038/s41375-019-0628-0 (PMC7214272; doi:10.1038/s41375-019-0628-0)
Supplement: Supplementary file 1 — Supplemental Material [file 41375_2019_628_MOESM1_ESM.docx]

**Supplementary Data**

**Supplementary Methods**

**Cell lines and treatment**

BL cell lines (Ramos, BL-41, Namalwa, Daudi, Jiyoye, Raji) and DLBCL cell lines (BJAB, SU-DHL-5, WSU-NHL, OCI-Ly1, WSU-DLCL2, Karpas-422, HT, OCI-Ly19, SU-DHL-4 and DoHH2) were cultured in RPMI1640 medium (Life Technologies) supplemented with 15 % fetal calf serum (PAN-Biotech, Aidenbach, Germany), 2 mM L-glutamine, 100 U/mL penicillin and 100 µg/mL streptomycin, and 50 µM monothioglycerol at 37°C and 5 % CO_2_.

Cell lines were authenticated by short tandem repeat (STR) DNA typing using GenomeLab^TM^ GeXP Genetic Analysis System (Sciex, Darmstadt, Germany) and GenomeLabHuman STR primer set (Beckman Coulter, Brea, CA, USA) or experiments were performed right after receipt from DSMZ. STR profiles were analyzed using the “Online STR Analysis” tool provided by DSMZ (comprehensive DSMZ database of STR cell line profiles, [www.dsmz.de](http://www.dsmz.de)) and the ExPASy bioinformatics resource portal database “Cellosaurus” (<https://web.expasy.org/cellosaurus/>; RRID: SCR_013869).

Mycoplasma contamination was controlled using the Mycoplasma Detection Kit-Quick-Test (Biotool.com, Absource Diagnostics, Munich, Germany).

**Vectors**

**shRNA-mediated knockdown**

The shRNA target sequences for *AKT1* ([1-5](#_ENREF_1)) and *PTEN* were selected from Broad Institute RNAi consortium shRNA library (https://www.broadinstitute.org/rnai-consortium/rnai-consortium-shrna-library). As a negative control we used a scramble sequence that does not have targets in the human genome ([6](#_ENREF_6)). Corresponding oligonucleotides were assembled and cloned into the BsbI and EcoRI cutting sites of pRSI12-U6-sh-UbiC-TagRFP-2A-Puro (Cellecta, <https://www.cellecta.com/>) using the Rapid DNA Ligation Kit (Roche, Basel Switzerland). ShRNA target sequence as well as amplification primers are listed in the table below.

| Vector | Broad Institute Clone ID | Target sequence | Amplification primers |
| --- | --- | --- | --- |
| pRSI12- pRSI12-AKT1sh | TRCN0000010162 | GGACAAGGACGGGCACATTAA | for-5´-ACCGGGGACAAGGACGGGCACATTAAG  TTAATATTCATAGCTTAATGTGCCCGTCCTTGTCCTTTTTTG  rev-5´-AATTCAAAAAAGGACAAGGACGGGCAC  ATTAAGCTATGAATATTAACTTAATGTGCCCGTCCTTGTCCC |
| pRSI12-PTENsh#1 | TRCN0000230371 | ATTTCGGGCACCGCATATTAA | for-5´-ACCGGATTTCGGGCACCGCATATTAAG TTAATATTCATAGCTTAATATGCGGTGCCCGAAATTTTTTTG  rev-5´-AATTCAAAAAAATTTCGGGCACCGCAT ATTAAGCTATGAATATTAACTTAATATGCGGTGCCCGAAATC |
| pRSI12-PTENsh#2 | TRCN0000002749 | CCACAAATGAAGGGATATAAA | for-5´-ACCGGCCACAAATGAAGGGATATAAAG TTAATATTCATAGCTTTATATCCCTTCATTTGTGGTTTTTTTG  rev-5´-AATTCAAAAAAACCACAAATGAAGGGA TATAAAGCTATGAATATTAACTTTATATCCCTTCATTTGTGGC |

**Expression vectors**

For gene expression we used the SF-LV vector ([7](#_ENREF_7)) (provided by K.L. Rudolph, Leibniz Institute for Age Research, Germany). For gene expression ORFs of genes of interest were cloned into the NotI cutting site of SF-LV-cDNA-EGFP by the Gibson Assembly method (NEB). Template vectors as well as amplification primers are listed in the table below.

| Vector | Template vector | Amplification primers |
| --- | --- | --- |
| SF-LV-myrAKT | pcDNA3-Myr-HA-Akt1 (gift from William Sellers, (Addgene plasmid #9008) | for-5´-TTAACTCGAGTTAACGCCCCACCACCATG GGGAGC  rev-5´-GGAGGGAGAGGGGGCTTAGGCCGTGCTGC TGGC |
| SF-LV-PTEN | pcDNA3-HA PTEN (gift from Jaewhan Song, Addgene plasmid #78776) | for-5´-TAATTAACTCGAGTTAACGCACCATGACAGCCATCATC  rev-5´-TTAGGGGGGGGGGAGGGAGAGGGGGCTCAGACTTTTGTAATTTGTGTATG |

**Immunoblotting**

Cell lines were lysed in Laemmli buffer containing 6 M urea and 5 % 2-mercaptoethanol or SDS sample buffer (62.5 mM Tris-HCl (pH 6.8), 2 % SDS, 10 % Glycerin, 50 mM DTT, 0.01% bromphenol blue) and boiled for 5 - 10 minutes at 100 °C. For detection of phosphorylated proteins, lysates were sonicated 3 times for 5 seconds before boiling. Lysates were separated by SDS-PAGE and electrophoretically transferred to a 0.45 µm nitrocellulose membrane. Membrane was blocked for 15 minutes at 37 °C in 5 % non-fat dried milk/TBS and primary antibodies were incubated at 4 °C overnight. Membrane was washed twice in TBS-T (0.5% Tween-20) and once in TBS (5 minutes per washing step). Horseradish peroxidase-conjugated secondary antibody was diluted 1:5000 - 1:10000 (goat anti-rabbit, #31460, Thermo Fisher Scientific, Waltham, MA, USA; RRID: AB_228341) or 1:5000 (goat anti-mouse: 1:10 000, #sc-2005, Santa Cruz Biotechnology, Dallas, TX, USA; RRID: AB_631736) in 5 % non-fat dried milk/TBS-T or TBS-T only and membrane was incubated for 1 h at RT. Washing of the membrane was repeated as before and protein bands were visualized by addition of SuperSignal West Dura Extended Duration Substrate (Thermo Fisher). TUBB, GAPDH, or RELA expression levels were used as loading controls.

| Primary antibody | Species | Product number | Dilution factor | RRID |
| --- | --- | --- | --- | --- |
| pAKT-T308 | rabbit | #13038, Cell Signaling | 1:1000 | AB_2629447 |
| pAKT-S473 | rabbit | #9271, Cell Signaling | 1:1000 | AB_329825 |
| pan-AKT | rabbit | #9272, Cell Signaling | 1:1000 | AB_329827 |
| PTEN | rabbit | #9552, Cell Signaling | 1:1000 | AB_10694066 |
| pRELA | rabbit | #3033, Cell Signaling | 1:1000 | AB_331284 |
| RELA | rabbit | #sc-372, Santa Cruz | 1:1000 | AB_632037 |
| MYB | rabbit | #12319, Cell Signaling | 1:1000 | AB_2716637 |
| CXCR4 | rabbit | #124824, Abcam | 1:500 | AB_10975635 |
| FOXO1 | rabbit | #2880, Cell Signaling | 1:250 | AB_2106495 |
| pFOXO1-T24 | rabbit | #9464, Cell Signaling | 1:1000 | AB_329842 |
| TUBB | rabbit | #6046, Abcam | 1:100000 – 1:800000 | AB_2210370 |
| GAPDH | rabbit | #25778, Santa Cruz | 1:1000 | AB_10167668 |

**Quantitative reverse-transcription PCR (qRT-PCR)**

Isolation of RNA, cDNA generation and RT-qPCR were performed as described previously ([8](#_ENREF_8)). For ensuring target specificity, primers were analyzed with Primer-BLAST (<http://www.ncbi.nlm.nih.gov/tools/primer-blast/>; RRID: SCR_003095) and subsequently synthesized by biomers.net. Annealing temperature was set as 60°C and samples were measured in duplicates using the LightCycler 480 instrument (Roche). The relative expression of the target gene was calculated with help of the 2^-ΔΔCT^ method. RPL13A was used as housekeeping gene. All primer sequences are listed below.

| **RT-qPCR primers** | |
| --- | --- |
| RPL13A | for-5´-CGGACCGTGCGAGGTAT  rev-5´-CACCATCCGCTTTTTCTTGTC |
| AICDA | for-5´-GCATGGTCACCTTCAAGCTA  rev-5´-TTGCGTTTCCAGAAGATTTG |
| RAD51 | for-5´-GGTGAAGGAAAGGCCATGTA  rev-5´-CATCACTGCCAGAGAGACCA |
| RAD51AP1 | for-5´-GACTTCGGTGGACTCTGCTC  rev-5´-GGTGGGACCCATTTAGGTTT |
| BACH2 | for-5´-TGGACAACTTCTCCTGCCTT  rev-5´-GGTCTGAGGACAGGGCAATA |
| MYB | for-5´-ACCATTGCCGACCACACCAG  rev-5´-TGGCGAGGCGCTTTCTTCAG |

**Lentiviral transduction**

HEK293T cells were transfected with the lentiviral plasmid of interest, the HIV-1 derived packaging plasmid p8.91 and the plasmid encoding the VSV-G envelope glycoproteins with help of polyethylenimine (PEI, Polysciences, Hirschberg an der Bergstraße, Germany). Viral supernatant was harvested 48 h and 72 h later and supplemented with 1 µg/ml polybrene. Cells were resuspended in viral supernatant and spinoculated at 2900 rpm for 2 h at 4 °C. Analysis was performed 3-5 days later.

**Growth dynamics measurement by flow cytometry analysis**

Flow cytometry for measurement of growth dynamics was performed using the FACSCanto (BD Biosciences). Percentages of GFP^+^ or RFP^+^ cell populations were analyzed using the BD FACSDiva Software (RRID:SCR_001456) or Flow Jo Software (RRID: SCR_008520). For growth dynamics experiments, the percentage of transduced cells was measured every 3 days. First measurement was performed 4 - 5 days post transduction and the percentage of transduced cells was set as 100 %.

**Cell surface staining**

1 x 10^6^ cells were washed with wash buffer (PBS, 0.5 % BSA, 2 mM EDTA) and resuspended in 100 µl wash buffer containing a 1:20 dilution of CXCR4-APC antibody (#17999941, Thermo Fisher; RRID: AB_1724115) or IgG2ak-APC isotype control (#17472441, Thermo Fisher; RRID: AB_10598641). After an incubation for 10 minutes at 4 °C in the dark, cells were washed with wash buffer and analyzed by flow cytometry (FACSCanto, BD Biosciences). Dotplots and histograms were created using the Flow Jo Software (RRID: SCR_008520).

**Cell viability analysis**

Drug sensitivities were determined by cell counting with help of the trypan blue dye exclusion method using the Vi-CELL XR cell viability analyzer (Beckman Coulter) or by the cell viability MTT assay. For MTT assays, cells were seeded in triplicates into 96 well plates at a density of 1 x 10^5^ cells per well in complete medium. Cells were treated with 2-fold serial dilutions of AZD5363 (#TMO-T1920, TargetMol, Boston, MA, USA), with the highest concentration of 20 µM. Solvent control wells were incubated with dimethyl sulfoxide (DMSO), with volume corresponding to highest drug treatment. Positive control wells were treated with 5 µg/ml puromycin (#540222, Merck). Cells were incubated at standard conditions for 5-6 days, followed by addition of 25 µl of the 5 mg/ml MTT solution (Thiazolyl Blue Tetrazolium Bromide, Sigma-Aldrich) and incubation for 2 hours at 37 °C. Then, 100 µl lysis buffer (20% SDS, 50% dimethylformamide, 2 % acetic acid, 0.15 mM HCl, pH 4.7) was added and after an overnight incubation at 37 °C, the optical densities (OD) were measured at 570 nm wavelength using the SpectraMax 250 microplate reader (Molecular Devices, San Jose, CA, USA) with help of the SoftMax Pro 3.0 software (Molecular Devices; RRID: SCR_014240). Percentage of growth inhibition at a given drug concentration was calculated as (1 - OD_drug_ - OD_puromycin_) / OD_DMSO_ * 100. The half maximal inhibitory concentration was calculated using GraphPad Prism software (RRID: SCR_002798).

**Cell cycle analysis**

5 x 10^5^ cells were washed with PBS and fixed by adding 1 ml PBS and 3 ml ice-cold 70 % ethanol dropwise to the sample during vortexing. After incubation on ice for at least 1 h, cells were centrifuged, resuspended in PBS containing 40 μg/mL PI (Sigma-Aldrich) and 100 μg/mL RNAseA (Amersham Pharmacia, Piscataway, NJ, USA) and incubated for 30 min at 37 °C. Cell cycle analysis was performed by flow cytometry using a FACSCalibur flow cytometer (BD Biosciences) and ModFit LT Version 2.0 software (Verity Software House, Topsham, ME; RRID: SCR_016106).

**Immunohistochemistry (IHC)**

IHC and H&E stainings of formalin fixed and paraffin embedded samples were done according to standard procedures. Antibody clones are listed in the table below.

| **Antigen** | **Clone** | **Retrieval** | **Dilution** | **Company** |
| --- | --- | --- | --- | --- |
| pAKT-T308 |  | Microwave, TRS, pH 9 | 1:300 | Abcam, ab38449 |
| CD20 | L26 | Steamer, TRS, pH 6.1 | 1:500 | DAKO, M0755 |
| CD3 | F7.2.38 | Pressure cooker, citrate, pH 7 | 1:100 | DAKO, M7254 |
| CD34 | QBEnd10 | Steamer, TRS, pH 6.1 | 1:100 | DAKO, M7165 |
| CD68 | PG-M1 | Steamer, TRS, pH 6.1 | 1:100 | DAKO, M0876 |

Immunostaining for pAKT-T308 was done following the standard procedure of streptavidin/biotin labelled peroxidase using the Rabbit specific HRP/AEC (ABC) Detection IHC Kit (Abcam, Cambridge, UK). The primary antibody pAKT-T308 (Abcam, ab38449, RRID: AB_722678) was applied in a 1:300 dilution. Immunostained tissues were evaluated by an experienced pathologist (P.M.). A simple scoring system for staining intensities was set up as follows: no staining, -; weak staining, +; moderate staining, ++; strong staining, +++.

**Luciferase Assay**

Namalwa cells stably expressing a NF-κB-dependent luciferase reporter (3× κB.luc) containing three copies of the κB motif immediately upstream of the β-globin TATA box ([9](#_ENREF_9)) were transduced with lentiviral vectors expressing PTsh or scrambled control or myrAKT or SFLV EV control and FACS sorted 4 days post transduction.

1 x 10^6^ cells were washed with PBS and lysed in 100 µL lysis buffer (100 mM KPO4 (pH 7.4), 0.2 % Triton X-100). After incubation on ice for 5 minutes, 50 µl Luciferase-Assay buffer (20 nM Tricine, 1.07 mM MgCO_3_MgOH_2_x5H_2_0, 2.7 mM MgSO_4_, 0.1 mM EDTA, 30 mM DTT, 0.27 mM Coenzym A, 0.5 mM Luciferin, 0.53 mM ATP) was added followed by luminescence measurement on the Lumat LB 9507 tube luminometer (Berthold Technologies, Germany). Protein concentration at 280 nm was measured by Nanodrop 1000 spectrophotometer (Thermo Fisher Scientific, USA) and luciferase values were normalized to protein amount.

**Isolation of tonsillar GC DZ B cells**

Tonsillar GC DZ B cells were isolated from tonsils of 29-35 years old patients undergoing tonsillectomy at the Department of Otorhinolaryngology, Head and Neck Surgery, University of Ulm, Germany. The written informed consent was obtained.

For isolation of cells from tonsillar tissue, tonsils were minced with scissors and pressed through a stainless-steel sieve. Peripheral blood mononuclear cells (PMBCs) were isolated by density gradient centrifugation using Pancoll human 1.077g/ml (PAN-Biotech, Aidenbach, Germany). B cells were isolated from PMBCs using the MACS cell separation system and the B Cell Isolation Kit II, human (Miletenyi Biotec, Bergisch Gladbach, Germany), followed by depletion of naïve IgD^+^ cells using the anti-IgD-Biotin (Biolegend, San Diego, CA, USA) antibody and Streptavidin Microbeads (Miltenyi Biotec). Purity of the isolated cells was confirmed by staining with anti-CD19-PE (Immunotools, Friesoythe, Germany) and anti-IgD-PE/Cy7 (Biolegend) antibodies as described in section “Cell surface staining”. Tonsillar B cells were then stained with anti-CD38-PE/Dazzle, anti-CXCR4-PE and anti-CD86-FITC (Biolegend). CD38^hi^/CXCR4^hi^/CD86^lo^ cells representing GC DZ B cells were sorted using the S3e Cell Sorter (Bio-Rad).

**References**

1. Brolih S, Parks SK, Vial V, Durivault J, Mostosi L, Pouyssegur J, et al. AKT1 restricts the invasive capacity of head and neck carcinoma cells harboring a constitutively active PI3 kinase activity. BMC cancer. 2018 Mar 5;18(1):249. PubMed PMID: 29506489. Pubmed Central PMCID: PMC5836445. Epub 2018/03/07. eng.

2. Ko SC, Huang CR, Shieh JM, Yang JH, Chang WC, Chen BK. Epidermal growth factor protects squamous cell carcinoma against cisplatin-induced cytotoxicity through increased interleukin-1beta expression. PloS one. 2013;8(2):e55795. PubMed PMID: 23383347. Pubmed Central PMCID: PMC3562190. Epub 2013/02/06. eng.

3. Roccaro AM, Leleu X, Sacco A, Jia X, Melhem M, Moreau AS, et al. Dual targeting of the proteasome regulates survival and homing in Waldenstrom macroglobulinemia. Blood. 2008 May 1;111(9):4752-63. PubMed PMID: 18316628. Pubmed Central PMCID: PMC2343604. Epub 2008/03/05. eng.

4. Wu DW, Wu TC, Wu JY, Cheng YW, Chen YC, Lee MC, et al. Phosphorylation of paxillin confers cisplatin resistance in non-small cell lung cancer via activating ERK-mediated Bcl-2 expression. Oncogene. 2014 Aug 28;33(35):4385-95. PubMed PMID: 24096476. Epub 2013/10/08. eng.

5. Liu ZM, Tseng JT, Hong DY, Huang HS. Suppression of TG-interacting factor sensitizes arsenic trioxide-induced apoptosis in human hepatocellular carcinoma cells. The Biochemical journal. 2011 Sep 1;438(2):349-58. PubMed PMID: 21649584. Epub 2011/06/09. eng.

6. Sarbassov DD, Guertin DA, Ali SM, Sabatini DM. Phosphorylation and regulation of Akt/PKB by the rictor-mTOR complex. Science. 2005 Feb 18;307(5712):1098-101. PubMed PMID: 15718470.

7. Wang J, Sun Q, Morita Y, Jiang H, Gross A, Lechel A, et al. A differentiation checkpoint limits hematopoietic stem cell self-renewal in response to DNA damage. Cell. 2012 Mar 2;148(5):1001-14. PubMed PMID: 22385964.

8. Vogel MJ, Xie L, Guan H, Tooze RM, Maier T, Kostezka U, et al. FOXO1 repression contributes to block of plasma cell differentiation in classical Hodgkin lymphoma. Blood. 2014 Nov 13;124(20):3118-29. PubMed PMID: 25232062.

9. Maier HJ, Marienfeld R, Wirth T, Baumann B. Critical role of RelB serine 368 for dimerization and p100 stabilization. J Biol Chem. 2003 Oct 3;278(40):39242-50. PubMed PMID: 12874295.

**Supplementary Tables**

**Supplementary Table 1. Sensitivities of BL and GCB-DLBCL cell lines to pan-AKT inhibitors**

| Cell line | MK-2206 | GSK690693 |  |
| --- | --- | --- | --- |
| BL IC50 | | |  |
| BL-41 | 2.78 | 0.22 |  |
| BL-70 | no value | 0.04 |  |
| CA46 | 12.00 | 6.13 |  |
| Daudi | 2.42 | 10.40 |  |
| DG-75 | 2.58 | 146.00 |  |
| EB-3 | 82.80 | 43.70 |  |
| EB2 | 42.30 | 0.07 |  |
| GA-10 | 0.59 | no value |  |
| JiyoyeP-2003 | 22.40 | 96.30 |  |
| NAMALWA | 1.79 | 1.85 |  |
| Raji | 48.60 | 119.00 |  |
| Ramos-2G6-4C10 | 0.81 | 24.20 |  |
| ST486 | 19.70 | 13.20 |  |
| GCB-DLBCL IC50 | | | AKT^high^ |
| DB | 13.90 | 90.00 | no |
| DOHH-2 | 1.47 | no value | yes |
| Farage | 0.53 | 0.59 | unknown |
| HT | 0.76 | 7.94 | yes |
| KARPAS-422 | 0.56 | 0.22 | yes |
| OCI-LY-19 | 5.53 | 2.29 | no |
| OCI-LY7 | 0.08 | no value | no |
| RL | 3.00 | 0.79 | yes |
| SU-DHL-10 | 0.40 | 0.07 | yes |
| SU-DHL-16 | 0.45 | 0.04 | yes |
| SU-DHL-4 | 1.26 | 0.05 | yes |
| SU-DHL-5 | 0.37 | 0.06 | yes |
| SU-DHL-6 | 0.13 | 0.04 | yes |
| SU-DHL-8 | 39.60 | 5.36 | no |
| WSU-DLCL2 | 0.36 | no value | yes |
| WSU-NHL | 0.21 | 80.20 | no |

IC50 values of BL as well as GCB-DLBCL cell lines to pan-AKT inhibitors GSK690693 and MK-2206 were collected from the Genomics of Drug Sensitivity in Cancer database ([www.cancerrxgene.org](http://www.cancerrxgene.org)) ([10](#_ENREF_10)).

**Supplementary Figures and Legends**

**
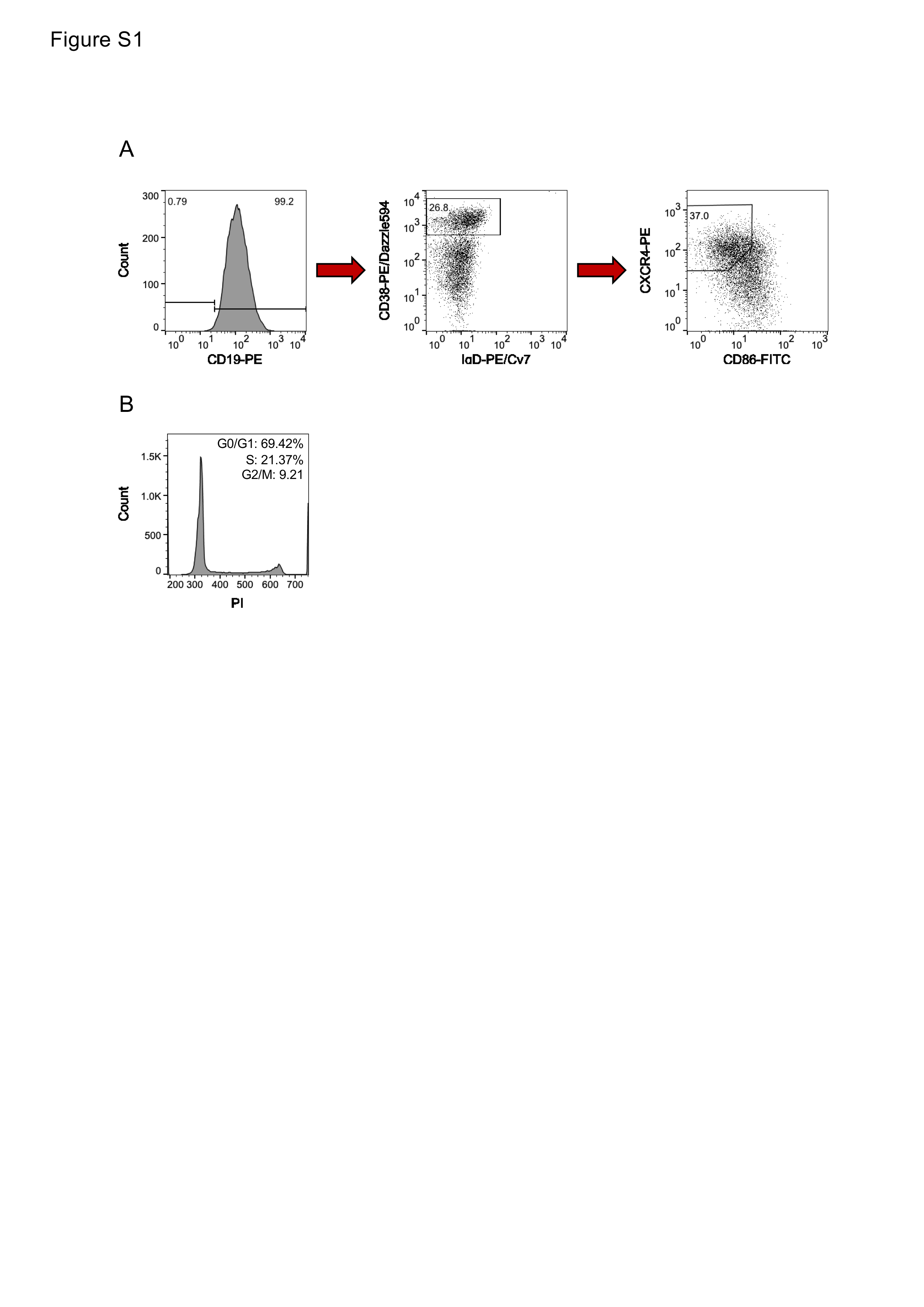
**

**Figure S1. Isolation strategy of tonsillar GC DZ B cells**

(**A**) Isolation strategy of tonsillar GC DZ B cells. For further information see Supplementary methods. (N=3) (**B**) Cell cycle analysis using PI DNA staining of FACS-sorted CD19^+^/IgD^-^/CD38^hi^/CXCR4^hi^/CD86^lo^ GC DZ B cells.

**
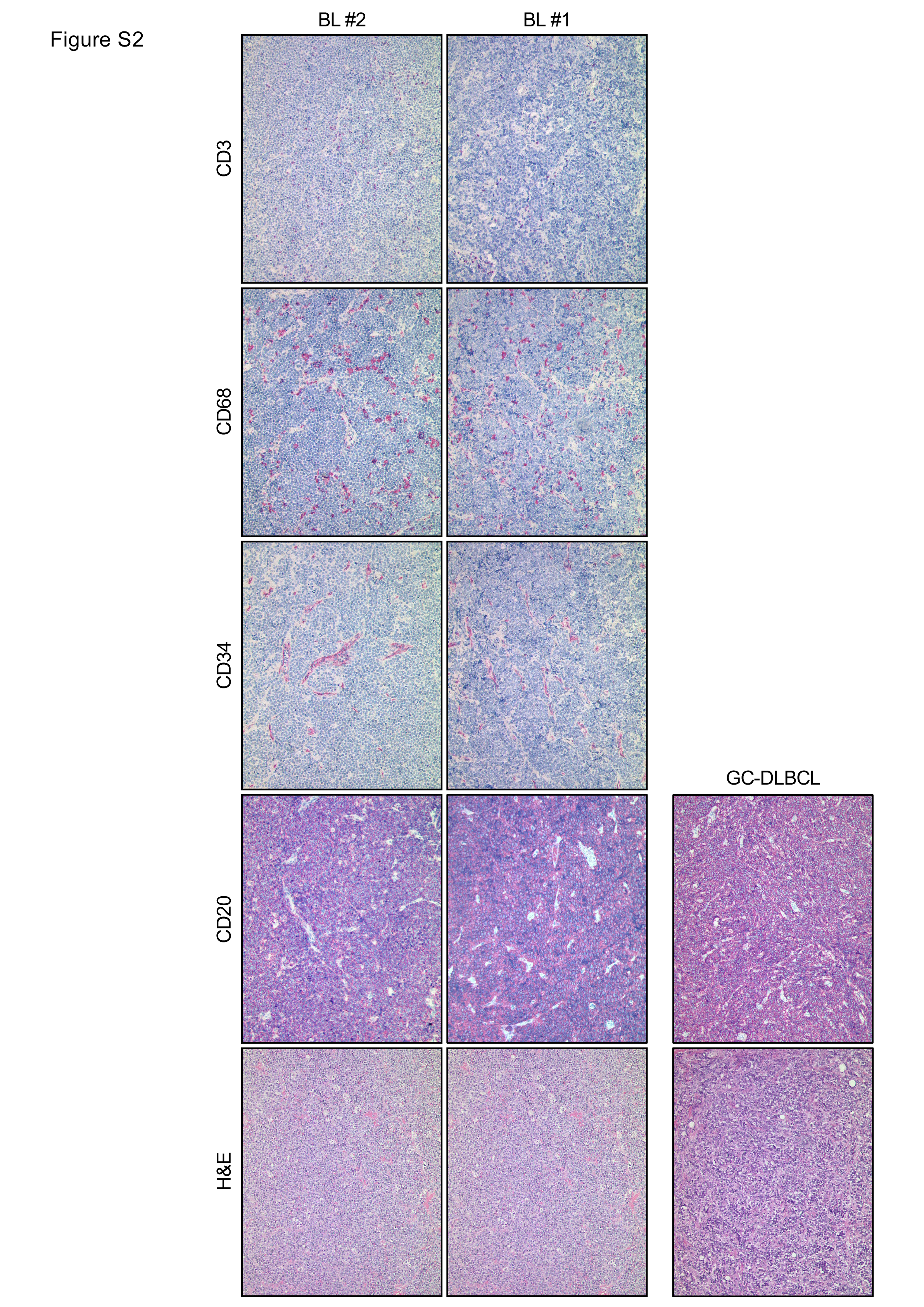
**

**Figure S2. Histology and immunohistochemistry of two typical BL and one GCB-DLBCL case**

The hematoxylin/eosin (H&E) staining of BL cases shows isomorphic medium-sized densely packed lymphoma cells with scattered intermingled tangible body macrophages. CD20 is expressed at the cell surface of neoplastic cells. Around 95 % of BL cells are CD20-positive. CD34 highlights the vascular endothelium accounting for less than 2 % of the cells composing BL. CD68 highlights the macrophage component accounting less than 2 % of the tumor volume. CD3 highlights the T-cell component of BL, which is minimal.


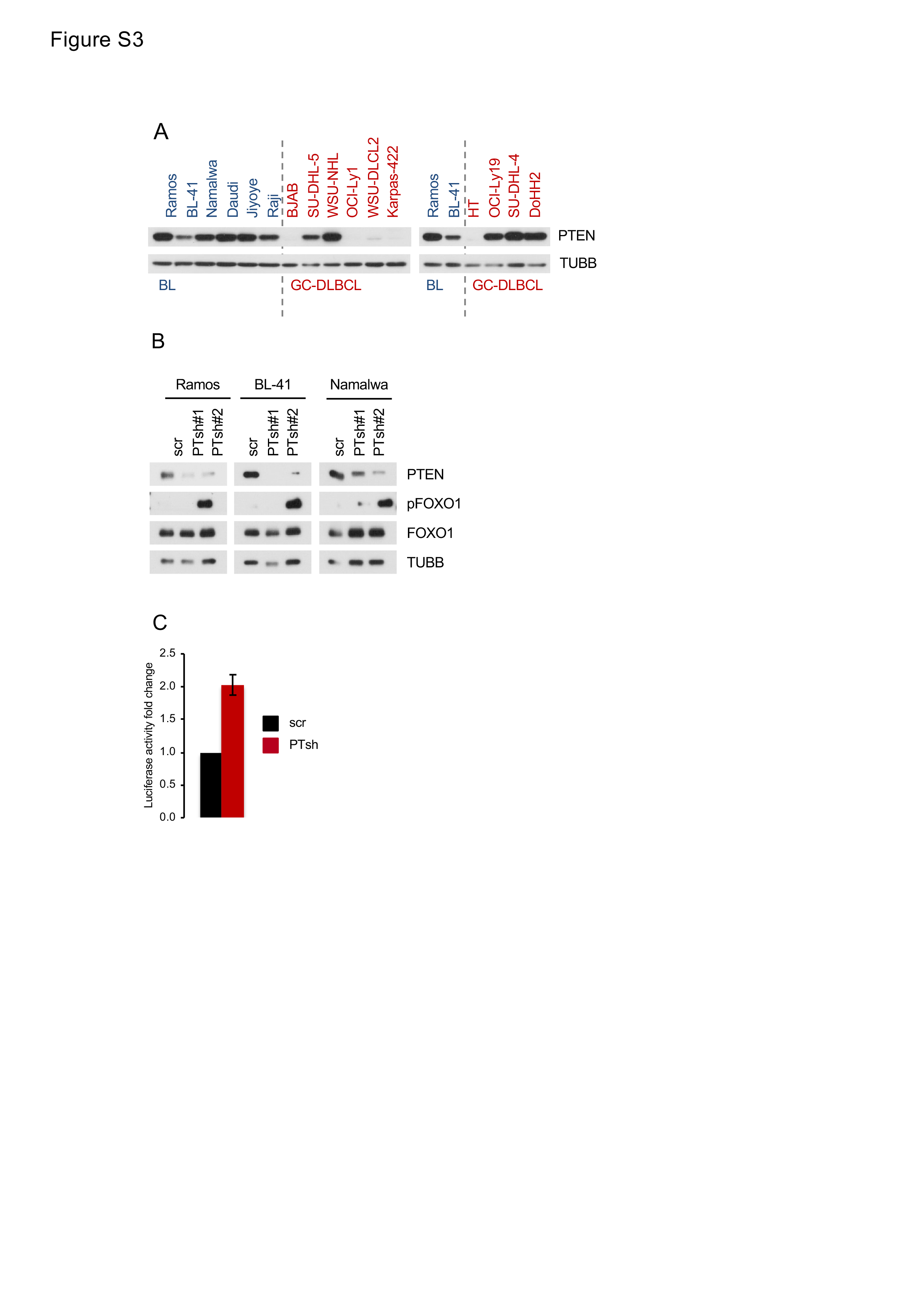
**Figure S3. PTEN expression in GCB-DLBCL and BL cell lines and effect of PTEN knockdown**

(**A**) Expression of PTEN in BL and GCB-DLBCL cell lines was analyzed by immunoblot. TUBB served as loading control. A representative of two independent experiments is shown. (**B**) PTEN knockdown results in upregulation of pFOXO1^T24^. BL cell lines were transduced with lentiviral plasmids expressing PTEN shRNAs (PTsh#1, PTsh#2) or scrambled control (scr). Transduced cells were FACS sorted 4 – 5 days post transduction and PTEN, FOXO1 and pFOXO1^T24^ levels were analysed using immunoblot. A representative of three independent experiments is shown. (**C**) Luciferase reporter assay. Namalwa cell line stably expressing a NF-κB-dependent luciferase reporter (3× κB.luc)([9](#_ENREF_9)) were transduced with a lentiviral vector expressing PTsh followed by FACS sorting for RPF 4 days post transduction. Luminescence was measured as described in Supplementary Methods. Data are shown as mean ± SD (N=3).
